# Bulk and single-cell transcriptome profiling reveals the dynamic immune response in granulomatous amebic encephalitis caused by *Balamuthia mandrillaris*: a cohort study

**DOI:** 10.3389/fimmu.2025.1677014

**Published:** 2025-10-02

**Authors:** Jingjia Zhang, Xinmiao Jia, Bin Yang, Ying Ge, Wei Jiang, Yang Cong, Huanwen Wu, Huifang Liu, Jingjing Tian, Yingchun Xu, Qiwen Yang

**Affiliations:** ^1^ Department of Clinical Laboratory, State Key Laboratory of Complex Severe & Rare Diseases, Peking Union Medical College Hospital, Peking Union Medical College, Chinese Academy of Medical Sciences, Beijing, China; ^2^ Clinical Biobank, Biomedical Engineering Facility of National Infrastructures for Translational Medicine, Chinese Academy of Medical Sciences, Peking Union Medical College Hospital, Beijing, China; ^3^ Center for Bioinformatics, National Infrastructures for Translational Medicine, Institute of Clinical Medicine & Peking Union Medical College Hospital, Chinese Academy of Medical Sciences and Peking Union Medical College, Beijing, China; ^4^ Vision Medicals Center for Infection Diseases, Guangzhou, China; ^5^ Department of Infectious Diseases, Peking Union Medical College Hospital, Chinese Academy of Medical Sciences and Peking Union Medical College, Beijing, China; ^6^ Medical Intensive Care Unit, Peking Union Medical College Hospital, Peking Union Medical College and Chinese Academy of Medical Sciences, Beijing, China; ^7^ Department of Pathology, State Key Laboratory of Complex Severe and Rare Diseases, Molecular Pathology Research Center, Peking Union Medical College Hospital, Chinese Academy of Medical Sciences and Peking Union Medical College, Beijing, China; ^8^ Biomedical Engineering Facility of National Infrastructures for Translational Medicine, Peking Union Medical College Hospital, Chinese Academy of Medical Sciences and Peking Union Medical College, Beijing, China

**Keywords:** *Balamuthia mandrillaris*, granulomatous amebic encephalitis, bulk RNA-sequencing, single-cell RNA-sequencing, differentially expressed genes, signaling pathway

## Abstract

**Introduction:**

Balamuthia mandrillaris infection in humans is rare and usually fatal. It is well established that *B. mandrillaris* can cause granulomatous amebic encephalitis (GAE). However, little is known about the factors that determine *B. mandrillaris* pathogenicity and its host-specific interactions.

**Methods:**

In this study, we conducted a cohort study of five patients with *B. mandrillaris* GAE and analyzed immune pathway alterations, differentially expressed genes (DEGs), and changes in immune cell composition to delineate the immune response in this rare infectious disease using bulk transcriptome analysis. Notably, we conducted bulk and single-cell transcriptome sequencing on paired cerebrospinal fluid (CSF) and blood samples from a single patient across three distinct stages of infection.

**Results:**

Analysis of seven CSF specimens revealed a total of 5,177 DEGs as the infection progressed. The most enriched pathway among the upregulated DEGs was the “PI3K-Akt signaling pathway”. Moreover, its upstream pathways, the “Toll-like receptor (TLR) signaling pathway” and “JAK/STAT signaling pathway”, were also upregulated. Among these, *TLR2, TLR9*, and *IFN-I/III*-related genes played a crucial role in activating these pathways. The single-cell transcriptome results served to validate these findings.

**Disscusion:**

Overall, our study aimed to elucidate the pathogenic mechanisms underlying *B. mandrillaris* GAE, providing novel insights into the associated immune responses.

## Introduction

*Balamuthia mandrillaris* infection in humans is rare and typically fatal, with fewer than 300 cases reported worldwide (1). In China, most patients initially present with skin lesions, followed by encephalitis several years later. Factors contributing to the low survival rate include delayed or missed diagnosis, lack of effective treatments, and the severity of infection (2). Raising awareness of this infection may facilitate earlier diagnostic and therapeutic interventions, potentially improving clinical outcomes (3). Therefore, prompt recognition and accurate diagnose of this condition are crucial. However, little is known about the factors that determine *B. mandrillaris* pathogenicity and the host factors influencing it (1). This knowledge gap has hindered the development of both diagnostic methods and effective treatments.

*B. mandrillaris* has been shown to induce the production of interleukin (IL)-6, a pleiotropic cytokine involved in the early stages of inflammation, in human brain microvascular endothelial cells (HBMECs) (4, 5). Through activation of phosphatidylinositol 3-kinase and serine/threonine protein kinase Akt, IL-6 increases blood–brain barrier (BBB) permeability, facilitating *B. mandrillaris* crossing (6). During clinical therapy, empirical anti-inflammatory steroids were administered to reduce central nervous system (CNS) inflammation (3). However, reports indicate that suppressing immune effectors may promote hematogenous spread of the amebae. Further clinical data are needed to determine whether host innate or adaptive immunological changes contributed to the patient’s decline after immunomodulatory therapy. Cerebrospinal fluid (CSF) was collected to aid diagnosis and to better understand neurological illnesses, as it represents a distinct immunological compartment surrounding the CNS. The leukocyte composition in CSF is tightly regulated and differs significantly from that in blood, whereas the noncellular portion of CSF mostly consists of an ultrafiltrate of serum. For instance, myeloid and B cells are reduced compared to blood, while CD4+ T lymphocytes are the most abundant cell type in CSF. Applying modern technologies to CSF, such as single-cell transcriptomics or bulk transcriptome analysis, enhances its ability to provide insights into the pathophysiology of neurological diseases, as demonstrated in studies of multiple sclerosis and Alzheimer’s disease.

In this study, we constructed the dynamic transcriptional profiles of CSF and revealed changes in gene expression, pathways, and immune cell populations triggered by *B. mandrillaris* GAE using bulk and single-cell transcriptome sequencing. Dynamic RNA-seq of CSF provides valuable insights into the inflammatory status of the CNS. Although our findings may represent only the “tip of the iceberg” regarding *B. mandrillaris* GAE-related alterations, they enhance our understanding and provide a foundation for future research and therapeutic interventions targeting this rare infectious pathogen.

## Materials and methods

### Enrolled cases and sample preparation

In this study, we successfully collected seven CSF specimens from five patients diagnosed with *Balamuthia mandrillaris* GAE. The first patient (P1) was enrolled at Peking Union Medical College Hospital (PUMCH). Magnetic resonance imaging (MRI) and immunohistochemistry confirmed a diagnosis of granulomatous encephalitis. P1 was further diagnosed with *B. mandrillaris* GAE by metagenomics next-generation sequencing (mNGS). Three CSF samples were collected from P1 on 23 October, 01 November, and 29 November 2021 (sample IDs: P1-CSF1, P1-CSF2, and P1-CSF3, respectively). Three blood samples were also collected from P1 on 31 October, 22 November, and 01 December 2021 (sample numbers were P1-B1, P1-B2, and P1-B3). The CSF sample P1-CSF3 was used for single-cell RNA sequencing (scRNA-seq). Additionally, CSF samples from four other *Balamuthia mandrillaris* GAE patients were collected. All four patients were diagnosed with GAE by mNGS and were from Guangdong, Shanxi, Hunan, and Guangdong Province, respectively (**Supplementary Table S7**). All CSF samples from five patients were used for bulk RNA-seq. With the exception of the P1-CSF3 sample, which was processed immediately for single-cell RNA sequencing, all other specimens were preserved in RNAlater and stored at − 80°C. The study protocols were approved by the Human Research Ethics Committee of PUMCH (approval number: JS-3347).

### Metagenomic next-generation sequencing

DNA was extracted from 300 μL CSF using the TIANamp Micro DNA Kit (DP316, TIANGEN BIOTECH, Beijing, China) following the manufacturer’s instructions. A negative “no template” control, consisting of elution buffer, was included to monitor potential contamination (7). The extracted DNA samples were then used for library construction. Libraries were prepared using transposase-mediated methods (Vision Medicals, Guangzhou, China). The quality of the DNA libraries was assessed using a Qsep1 Bio-Fragment Analyzer (BiOptic Inc., La Canada Flintridge, CA, USA) to evaluate adapter presence and fragment sizes prior to sequencing. Qualified libraries had fragment sizes of 300~500 bp, free of adapters and polymerase chain reaction (PCR) dimers, with concentrations greater than 0.5 ng/μL. Finally, the qualified DNA libraries were pooled and sequenced on the NextSeq 550 Dx platform (Illumina, San Diego, CA, USA) (8).

### Cytokine detection

Prior to cryopreservation, the specimens were tested for cytokines. CSF samples were centrifuged at 12,000 rpm for 10 min at 2°C–8°C. Subsequently, 0.5 mL of the supernatant was aliquoted and analyzed according to the assay kit instructions (Wellgrow Technology, Changsha, China). Cytokine detection was based on the principle of double-antibody sandwich flow fluorescence luminescence technology.

### Bulk RNA sequencing

Aliquots of 200 μL from each CSF and plasma sample of two patients were lysed in Trizol LS (Thermo Fisher Scientific, Carlsbad, CA, USA), followed by RNA extraction using a Direct-zol RNA Miniprep Kit (Zymo Research, Irvine, CA, USA) according to the manufacturer’s instructions. Purified RNA (10 μL) was used for cDNA generation and library preparation using an Ovation Trio RNA-Seq Library Preparation Kit (NuGen, San Carlos, CA, USA), following the manufacturer’s instructions. Bulk RNA-seq was then performed on an Illumina NextSeq 500 platform (Illumina, USA).

### Single-cell RNA sequencing

scRNA-seq libraries were prepared using the Chromium Single Cell 3′ Reagent Kits v3 (10X Genomics). Briefly, CSF cells were washed three times with 0.04% BSA in DPBS and resuspended to a concentration of 700~1,200 cells/μL (viability ≥ 85%). Cells were then captured in droplets at a targeted recovery rate. After reverse transcription, emulsions were broken, and barcoded cDNA was purified with Dynabeads, followed by PCR amplification. The amplified cDNA was subsequently used to construct 3′ gene expression libraries. For the construction of gene expression libraries, amplified cDNA (50 ng) was fragmented and end-repaired, followed by double-size selection using SPRIselect beads. Libraries were then sequenced on an NovaSeq 6000 platform (Illumina, USA) to generate 150 bp paired-end reads.

### Real-time PCR assay of *B. mandrillaris*

Primers for *B. mandrillaris* detection, RNase P/FW (5′-GGCAGGTTCCGAGGAGACA-3′) and RNase P/RV (5′-GTGGCCTTGTGTATTGAACTTAACATT-3′), were used as previously described (9). glyceraldehyde-3-phosphate dehydrogenase (GAPDH) was employed as an internal control to quantify the host cells using the primers GAPDH/FW (5′-GCACCGTCAAGGCTGAGAAC) and GAPDH/Rv (5′-TGGTGAAGACGCCAGTGGA). PCR reactions were carried out using SYBR^®^ Premix Ex Taq™ II (Takara, Beijing, China) in a total reaction volume of 50 μL, containing 0.4 μM of each primer and 0.1 μM of the probe. PCR runs were conducted on the AB7500 (Applied Biosystems, Foster City, CA, USA) using a hold at 95°C for 15 min, followed by 40 cycles of 95°C for 15 s (denaturation) and 60°C for 1 min (annealing/elongation). Fluorescence data were collected at the end of the annealing/elongation step. All experiments were performed three times, and PBS served as a negative control.

### Raw data processing and bioinformatics analysis

Briefly, the fastq files underwent adaptor trimming, removal of low-quality reads, and elimination of short reads using Fastp 0.20.0 (10). All clean data were mapped to the human genome GRCh38 using HISAT2 v2.1.0 with default parameters (11). Bam files were sorted by Samtools 1.9 (12). Gene counts were summarized using the featureCounts program within the Subread package release 2.0.0 (http://subread.sourceforge.net/) (13). To identify differentially expressed genes (DEGs) between the two groups, genes present in less than 50% of samples in both groups and genes with average counts per million (CPM) below five in both groups were excluded. The remaining gene counts were subsequently normalized using the normalization module in the Bioconductor DESeq2 package (14). DEGs were identified using the criteria of an adjusted *p*-value < 0.05 and an absolute logged fold-change (Log2FC) ≥ 2. For functional enrichment analysis of DEGs, KEGG pathway analysis (15) was conducted via KOBAS 3.0 (16). To estimate the composition of immune cells in CSF, raw gene counts were normalized to transcripts per million (TPM) and analyzed using the Cell-type Identification By Estimating Relative Subsets Of RNA Transcripts (CIBERSORT) algorithm v1.06 (17) with the original LM22 gene signature file and 1,000 permutations. Twenty-two immune cell subtypes were identified and further summarized into nine major immune cell types. Samples with a *p*-value < 0.05, reflecting the statistical significance of the deconvolution results across all cell subsets, were included. Raw data (raw reads) in fastq format were generated from the Raw BCL files using Illumina’s bcl2fastq converter. For these raw data, primary quality control was performed first. The monitored quality assessment parameters included: (i) containing more than three N bases; (ii) a proportion of bases with a quality value below 5 exceeding 20%; and (iii) presence of adapter sequences. All downstream analyses were conducted using high-quality clean data. The raw reads were demultiplexed and aligned to the reference genome using the 10X Genomics Cell Ranger pipeline (https://support.10xgenomics.com/single-cellgeneexpression/software/pipelines/latest/what-is-cell-ranger) with default parameters (18). Unless otherwise specified, all single-cell analyses were performed using Cell Ranger and Seurat. Briefly, for each gene and each cell barcode (filtered by Cell Ranger), unique molecular identifiers were counted to generate digital expression matrices. Secondary filtration was performed using Seurat (19). Genes expressed in more than three cells were considered as expressed, and each cell was required to contain at least 200 expressed genes. Foreign cells were filtered out. The Cell Ranger count pipeline processes fastq files by performing alignment, filtering, barcode counting, and UMI counting. It uses the Chromium cellular barcodes to generate feature barcode matrices through Cell Ranger count or Cell Ranger aggr, and subsequently reruns dimensionality reduction, clustering, and gene expression algorithms with default parameter settings. Secondary analysis of gene expression was carried out using Seurat. The Seurat package was used for data normalization, dimensionality reduction, clustering, and differential expression. The Seurat alignment method, canonical correlation analysis (CCA), was applied for integrated analysis of datasets. For clustering, highly variable genes were selected, and the principal components based on those genes were used to build a graph, which was segmented at a resolution of 0.6.

### Quantification and statistical analysis

For differential analysis, we then collected previously published scRNA-seq data from controls with idiopathic intracranial hypertension (IIH) (*n* = 9), a noninflammatory condition characterized by excess CSF, and from patients with the autoimmune brain disease multiple sclerosis (MS) in relapse (*n* = 9) (20, 21). The following inclusion criteria were applied to these MS/IIH patients: (1) treatment-naive patients with an episode suggestive of MS/IIH or with relapsing-remitting multiple sclerosis (RRMS) diagnosed according to MAGNIMS criteria, (2) absence of serious concomitant infectious diseases (e.g., HIV, meningitis, or encephalitis), and (3) absence of immunological comorbidities (e.g., rheumatic diseases). Fisher’s exact test was used for categorical variables, and the Mann–Whitney *U* test was used for continuous variables that do not follow a normal distribution. *p-*values from multiple testing were adjusted (*p*-value adjusted) using the Benjamini–Hochberg false discovery rate (FDR) with a significance level of 0.05 (22).

## Results

### Bulk transcriptome data reveal critical changes in gene expression and pathways

In order to describe host response in the CNS and blood of the immunocompetent GAE patient, we performed bulk RNA-seq of the entire CSF cohort. RNA-seq of CSF from a patient (43-year-old man) who was pathogen-negative (mNGS, IgG/IgM, Quantitative Real-time polymerase chain reaction (qPCR), and culture were negative) and diagnosed with noninfectious guided epilepsy was used as the negative control (NC). For RNA-seq, specimens from each time point had three technical replicates. More than 40 million reads were generated for each replicate. Among them, about 41.2% ± 23.5% of the reads could be mapped to the human genome and were used for further analysis.

**Figure 1:**
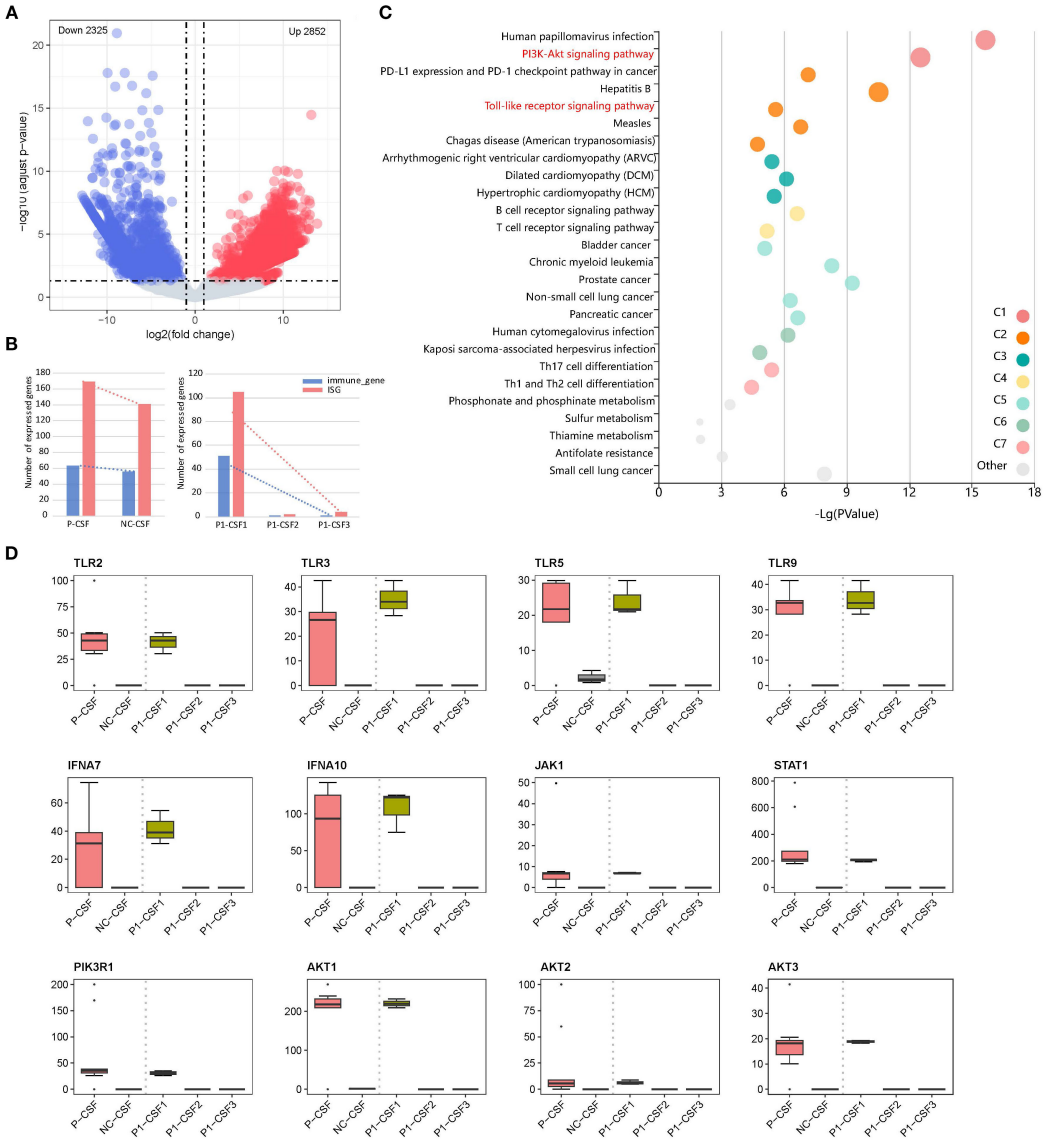
**(A)** Volcano plot of DEGS in CSF between patients and NC, showing 2,852 upregulated genes and 2,325 downregulated genes. **(B)** Changes in immune-related gene and ISG expression. **(C)** Pathway enrichment of upregulated DEGs between P-CSF and NC. **(D)** Changes in expression levels of upregulated gene (*TLR2*, *TLR3*, *TLR5*, *TLR9*, *IFNA7*, *IFNA10*, *JAK1*, *STAT1*, *PIK3R1*, *AKT1*, *AKT2*, and *AKT3*) associated with the “PI3K-AKT signaling pathway” and its upstream “Toll-like receptor (TLR) signaling pathway” and “JAK/STAT signaling pathway” among P1-CSF1, P1-CSF2, P1-CSF3, P-CSF, and NC. P-CSF represents the overall profile of seven CSF samples from five patients.

We analyzed the changes in seven CSF specimens from five patients compared with NC. An adjusted *p*-value (*q*-value < 0.05) and fold change (FC) ratio (|log2FC| ≥ 2) were used to determine the DEGs (**Supplementary Table S1**). We detected a total of 5,177 differentially expressed genes, of which 2,852 were upregulated DEGs and 2,325 downregulated DEGs in patients compared with NC (**Figure 1A**). In addition, we found that interferon-stimulated genes (ISG) and immune-related genes were highly expressed in P1-CSF1 and decreased after immunomodulatory treatment (**Figure 1B**). This could explain why the levels of major cytokines in the CSF of P1 gradually decreased (**Supplementary Table S2**).

We then performed pathway enrichment analysis of upregulated DEGs between seven CSF specimens and NC (**Figure 1C**). The “Phosphoinositide 3-Kinase–Protein Kinase B (PI3K-Akt) signaling pathway” was significantly enriched (*q*-value = 5.35*E*−11). The PI3K-Akt pathway is an important intracellular signal transduction pathway that responds to extracellular signals and promotes metabolism, proliferation, cell survival, growth, and angiogenesis (**Supplementary Figure S1**). Additionally, activation of PI3K-Akt signaling is a strategy employed by viruses to slow down apoptosis and prolong viral replication in both acute and persistent infections. From our results, it can also be seen that the “human papillomavirus infection” pathway was activated, indicating that infection with *B. mandrillaris* activates pathways similar to viral infections. To further understand how the PI3K-Akt signaling pathway is activated, we analyzed its upstream pathways. We found that the TLR signaling pathway and the “Janus Kinase–Signal Transducer and Activator of Transcription (JAK/STAT) signaling pathway” were also upregulated. In the TLR signaling pathway, *TLR2*, *TLR3*, *TLR5*, and *TLR9* were activated (**Supplementary Figure S2**). The JAK/STAT signaling pathway functions through *interferon (IFN)-I/III* (**Supplementary Figure S3**). After activation, PI3K-Akt signals are transmitted to downstream pathways, leading to the activation of several pathways, including protein translation, cell cycle, apoptosis, and p53 pathways. In our results, the Mitogen-Activated Protein Kinase (MAPK), Nuclear Factor Kappa-Light-Chain-Enhancer of Activated B cells (NFκB), and p53 signaling pathways were activated to perform downstream functions.

We further checked the expression of key factors in these pathways (*TLR2*, *TLR3*, *TLR5*, *TLR9*, *IFNA7*, *IFNA10*, *JAK1*, *STAT1*, *PIK3R1*, *AKT1*, *AKT2*, and *AKT3*), which were highly expressed at P1-CSF1, and found that they significantly decreased at P1-CSF2 or P1-CSF3 (**Figure 1D**). We also included the other four GAE patients and confirmed the pathway and gene expression pattern by bulk RNA sequencing of P-CSF. *TLR2* (*p* = 2.9*E*−07), *TLR3* (*p* = 8.0*E*−04), *TLR5* (*p* = 1.8*E*−03), *TLR9* (*p* = 3.7*E*−05), *IFNA7* (*p* = 6.6*E*−08), *IFNA10* (*p* = 1.2*E*−03), *JAK1* (*p* = 1.4*E*−05), *STAT1* (*p* = 5.8*E*−13), *PIK3R1* (*p* = 1.4*E*−08), *AKT1* (*p* = 2.7*E*−05), *AKT2* (*p* = 1.6*E*−04), and *AKT3* (*p* = 2.5*E*−03) were also highly expressed in P-CSF (**Figure 1D**). The IFN-stimulated JAK/STAT, TLR, PI3K-Akt, and downstream MAPK, NFκB, and p53 pathways contributed to the immune response against *B. mandrillaris*.

At the same time, we also noticed that pathways associated with CD4+ T cells were activated in”Th1 and Th2 cell differentiation” and “Th17 cell differentiation”. Th2 cells mediate immunity to parasites, and viral infections generally activate the production of Th1 cells (23, 24). Previous studies have demonstrated that CD4+ T cells play an important role in the immune response to *B. mandrillaris* infection (25). Three CSF specimens of P1 were also measured for cytokines. IL-6, IL-10, IFN-γ, and tumor necrosis factor alpha (TNF-α) had high secretions (**Supplementary Table S3**).

In addition to CSF, we also analyzed the bulk RNA-seq data of blood to detect the host response in blood. We detected a total of 3,665 DEGs, of which 1,707 were upregulated and 1,958 downregulated in P1-B1 compared to NC (**Supplementary Figure S4**). This result indicates that there is no strong response in the blood as observed in the CSF. The pathways in P1-B1 were also revealed, with upregulated “inositol phosphate metabolism” (*q*-value = 3.14*E*−05) and “phosphatidylinositol signaling system” (*q*-value = 0.00036), along with “epithelial cell signaling in *Helicobacter pylori* infection” (*q*-value = 0.00021) and “pathogenic *Escherichia coli* infection (*q*-value = 0.00037), which are related to bacterial invasion of epithelial cells. It can be seen that, unlike the response in CSF, which is similar to viral infections, the response in the blood resembles bacterial invasion. In addition, ISG and immune-related genes are highly expressed in P1-B1 and decrease after immunomodulatory treatment (**Supplementary Figure S4C**).

### Changes in immune cells during infection

**Figure 2:**
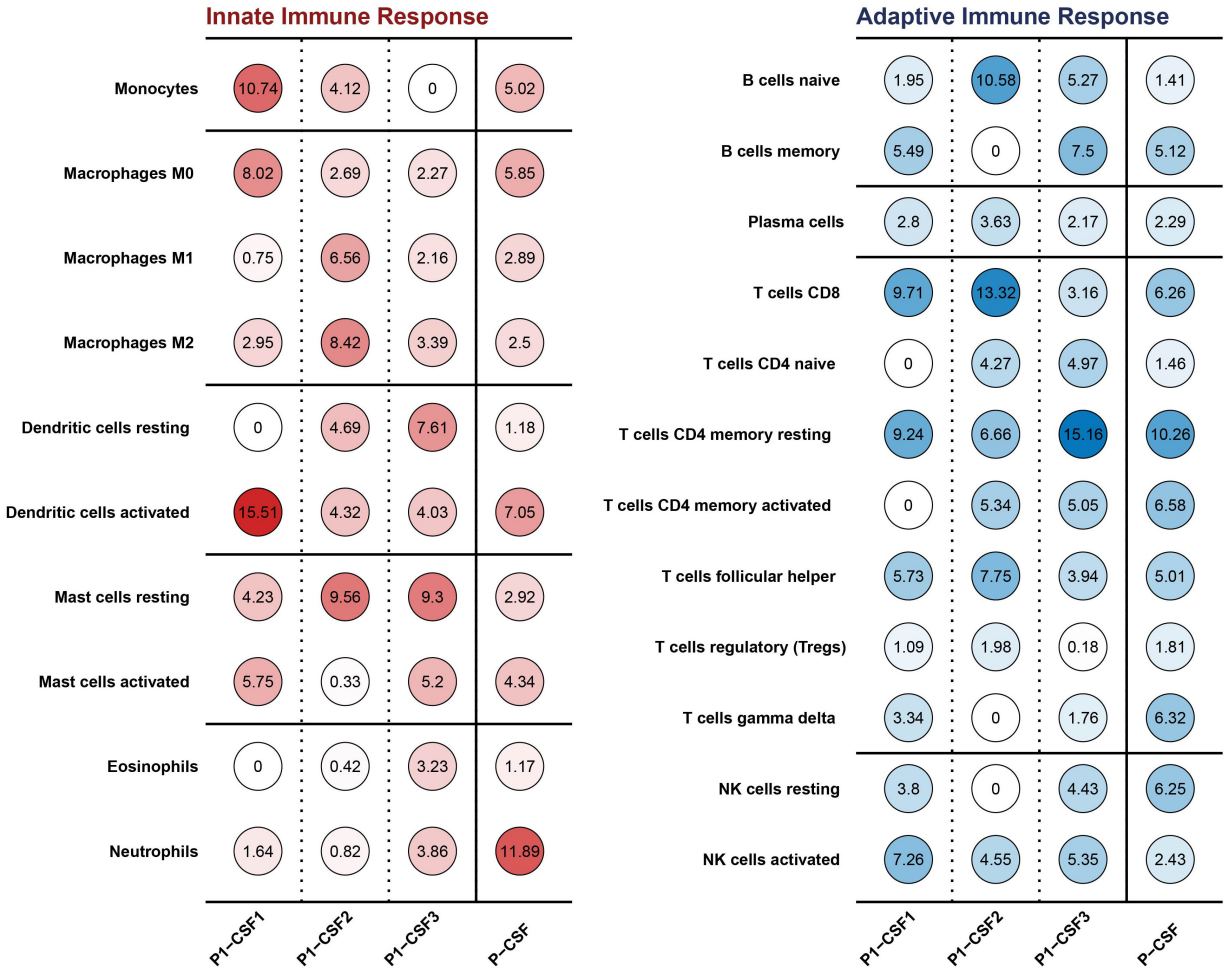
Innate and adaptive immune cell types and proportions in CSF among P1-CSF1, P1-CSF2, P1-CSF3, and P-CSF.

We analyzed the RNA-seq data of three CSF samples from P1 and estimated the type and proportion of various immune cells using CIBERSORT (26). Monocytes and activated dendritic cells were clearly depleted (**Figure 2**). After *B. mandrillaris* breaks through the blood–brain barrier, it initiates an early inflammatory response and recruits white blood cells to the site of infection, so the proportion of all kinds of white blood cells in P1-CSF1 is generally high (5). Dendritic cells were the most functional, full-time antigen-presenting cells, responsible for initiating, regulating, and maintaining the immune response. Similarly, monocytes act as important white blood cells. The depletion of these three types of cells can greatly exacerbate the infection. There was no clear pattern to the changes in immune cells in the blood (**Supplementary Figure S5**).

### Clinical information of the *Balamuthia mandrillaris* GAE patient

**Figure 3:**
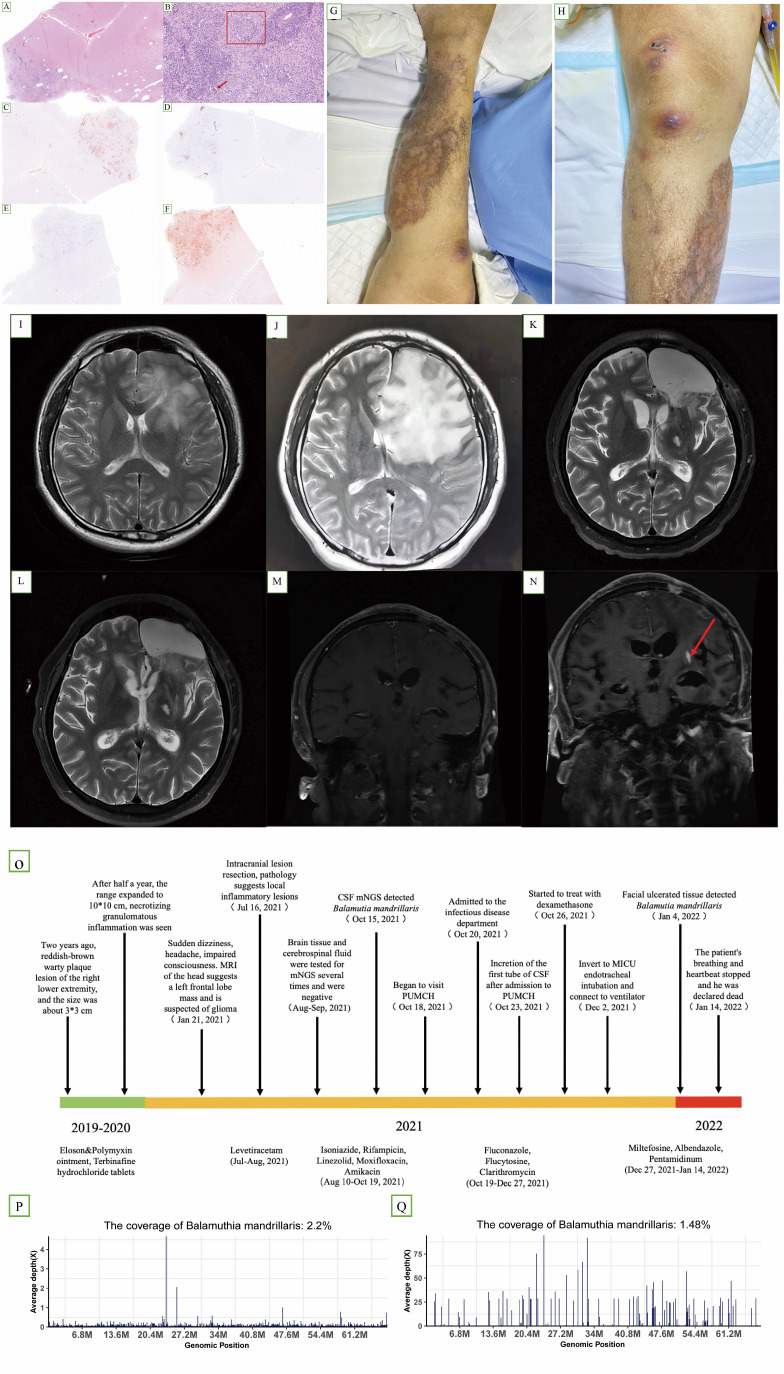
**(A–F)** Hematoxylin and immunohistochemistry staining of brain lesions revealed epithelial granuloma [**(B)** box] and multinucleated giant cell reaction [**(B)** arrow]. **(C, D, F)** Partially positive for CD3, CD20, and CD68, respectively. **(E)** Vascular tissue was positive for CD34. **(G, H)** Cutaneous lesion on the right lower extremity at later stages of the course. **(I)** Axial T2-weighted image (25 January 2021) revealed a space-occupying lesion in the left frontal lobe. **(J)** Axial T2-weighted image (13 July 2021), preoperative examination, showing that the lesion had enlarged; **(K**, **M)** axial T2-weighted image (15 November 2021); **(L, N)** axial T2-weighted image (8 December 2021). Compared with **(M, N)** showed an increase in lesions. **(O)** Timeline of the clinical course of the patient. **(P)** A total of 189,060 sequence reads out of 10,971,940 sequencing reads derived from the patient’s cerebrospinal fluid sample were mapped to the *B. mandrillaris* genome (15 October). **(Q)** A total of 41,413 sequence reads out of 15,804,200 sequencing reads derived from the patient’s facial tissue were mapped to the *B. mandrillaris* genome on 4 January.

P1 was an immunocompetent 37-year-old man with a reddish-brown warty plaque lesion on his right lower extremity for half a year (**Figures 3G, H, O**). MRI of the head revealed a space-occupying lesion in the left frontal lobe, which was suspected to be a glioma (**Figure 3I, J**). He underwent intracranial lesion resection on 16 July 2021. The pathology of brain tissue suggested local inflammatory lesions, perivascular infiltration of inflammatory cells, and a small amount of epithelial granuloma and multinucleated giant cell reaction (**Figures 3A, B**). After the operation, there was memory loss but was still able to take care of himself, and levetiracetam 0.5 g bid was given as antiepileptic treatment. He was then considered to have an intracranial infection, but the possibility of tuberculosis could not be ruled out. Antituberculosis and antibacterial therapy was given (isoniazide, rifampicin, linezolid, moxifloxacin, and amikacin) (**Figure 3O**).

As his condition worsened, he was admitted to the infectious disease department of PUMCH on 18 October 2021. MRI of the head showed abnormal enhancement foci in the left frontal lobe, consistent with infectious lesions (**Figures 3K, M**). MRI showed that the lesion was enlarged after one month (**Figure 3L, N**). Brain tissue immunohistochemistry analyses showed partial positivity for CD68, CD3, and CD20, and vascular tissue was positive for CD34 (**Figure 3C–F**). Fluconazole 400 mg quaque die (qd), flucytosine 1.5 g quater in die (qid), and clarithromycin 500 mg bis in die (bid) were administered for antiparasitic treatment. Later, miltefosine 50 mg quaque 8 hora per os (q8h po), albendazole 2# q12h po, and pentamidinum 300 mg intravenous (iv) qd were used. Dexamethasone was introduced on 26 October (**Figure 3O**). We separately collected three CSF samples on 23 October, 02 November, and 29 November 2021 (correspondingly, sample IDs were P1-CSF1, P1-CSF2, and P1-CSF3). Additionally, three blood samples were collected on 31 October, 22 November, and 01 December 2021 (sample ids were P1-B1, P1-B2, and P1-B3). In the untreated CSF (P1-CSF1), 189,060 microbial reads were specifically mapped to *B. mandrillaris* out of 10,971,940 sequencing reads (**Figure 3P**), with the mapped reads spanning the closest matched *B. mandrillaris* genome deposited in the NCBI nucleotide reference database. On 14 January 2022, the patient was declared dead. Before his death, similar lesions appeared on the facial skin. On 4 January 2022, we detected 41,413 microbial reads specifically mapped to *B. mandrillaris* out of 15,804,200 sequencing reads in facial tissue (**Figure 3Q**). Furthermore, we used qPCR to quantify the *B. mandrillaris* burden in CSF. Results showed that the parasite load increased over time compared with the host housekeeping gene GAPDH (**Supplementary Table S3**). Single-cell transcriptome further confirms cellular and functional changes in GAE CSF during deterioration.

**Figure 4:**
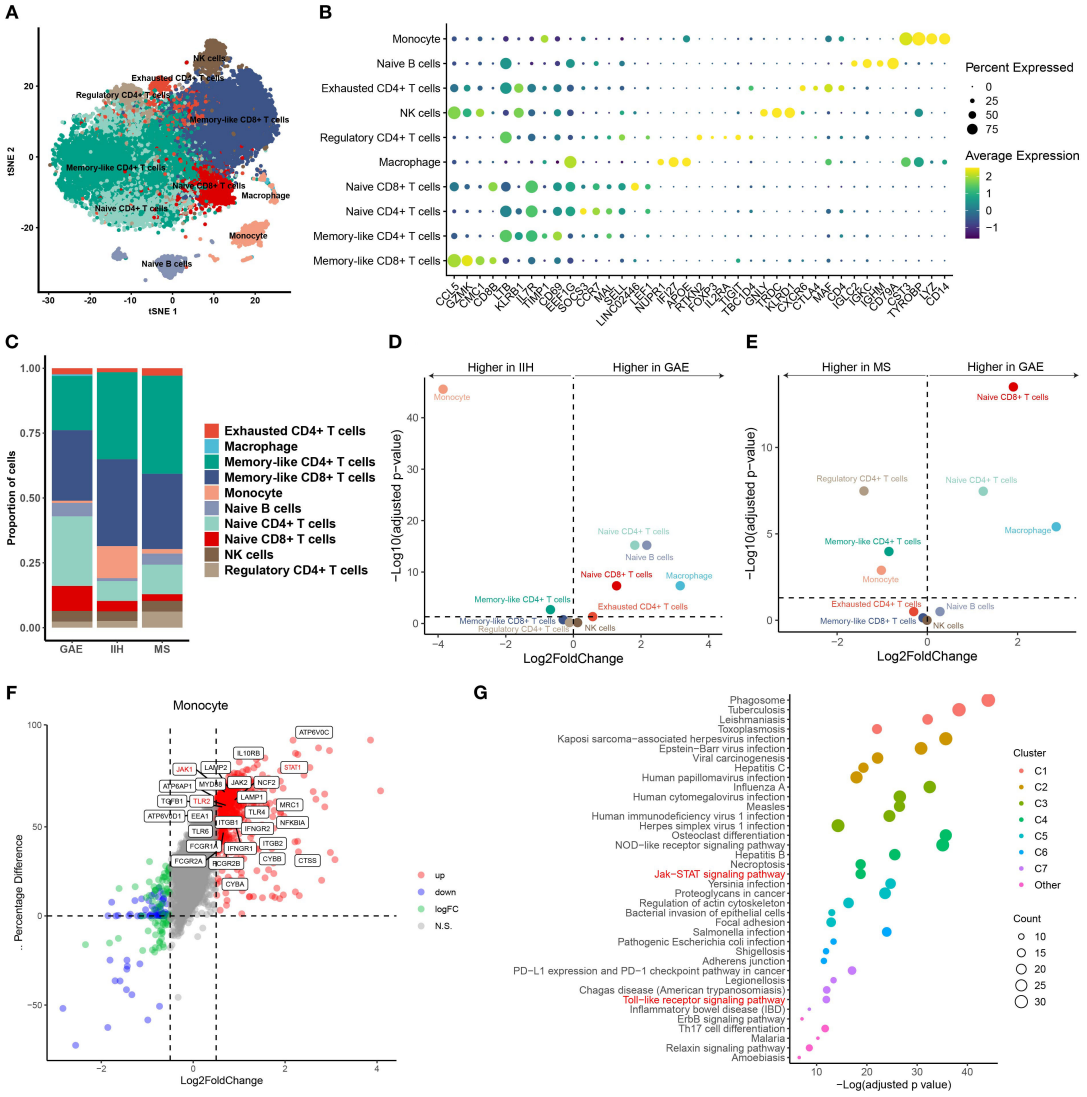
**(A)** Uniform Manifold Approximation and Projection (UMAP) plot showing 10 color-coded cell clusters of raw single-cell transcriptomes from CSF cells of GAE, IIH, and MS patients. T cells were dominant. **(B)** Marker genes of cell clusters are shown. Color encodes average gene expression; dot size represents the percentage of cells expressing the gene. **(C)** Proportions of cells split by diagnosis. **(D, E)** Changes in cluster abundances in GAE versus IIH **(D)** and GAE versus MS **(E)**. Logarithmic fold change of cluster abundance is plotted against the negative logarithmic *p*-value (two-sided Wilcoxon’s rank-sum test). The horizontal dashed line represents the significance threshold (*p* = 0.05). **(F)** Differential gene expression of monocytes between GAE and noninfectious controls (IIH, MS). *TLR2*, *JAK1*, and *STAT1* genes are still highly expressed in monocytes compared with IIE and MS after immunomodulatory treatment. **(G)** Functional enrichment analysis of monocyte upregulated genes between GAE and non-infectious control (IIH, MS). “Toll-like receptor (TLR) signaling pathway” and “JAK/STAT signaling pathway” remain highly enriched.

For further validation of our findings, we conduct the scRNA-seq analysis for P1-CSF3. Due to the difficulty in collecting CSF samples from healthy controls, we used previously published scRNA-seq data from controls with IIH and the autoimmune brain disease multiple sclerosis (MS) in relapse. We merged all available scRNA-seq data of GAE and control patients (IIH and MS), which encompassed 24,840 total single-cell transcriptomes with 8,280 ± 859 SEM average cells per sample and 1,024 ± 500 SEM median genes detected per cell (**Supplementary Table S6**). We performed unbiased cell clustering (**Figure 4A**) and annotated the resulting 10 clusters based on the expression of marker genes (**Figure 4B**; **Supplementary Table S6**). T leukocytes were dominant in CSF, even though our patient received immunomodulatory treatment (**Figure 4A**). T-cell subsets are transcriptionally similar and tend to require targeted analytical approaches. Therefore, we applied cell set enrichment analysis (CSEA) to identify cluster-independent compositions (27). This returned the following subclusters: naive CD8+ T cell clusters (*CD8B*, *CCR7*, *IL7R*, *LEF1*), memory-like CD 8+ T cells (*CD8B*, *CCL8*, *GZMK*, *CMC1*), naive CD4+ T cells (*CCR7*, *SELL*, *MAL*), and memory-like CD4+ T cells (*IL7R, CD69*) (**Figure 4B**). A further cluster, which we named exhausted CD4+ T cells, expressed T-cell exhaustion transcripts (*CTLA4*, *MAF*, *CXCR6*). NK cells (*KLRD1*, *GNLY*) clustered together with γδ T cells (*TRDC*) (**Figure 4B**). B lineage clusters were defined by *IGLC2*, *IGKC*, *IGHM*, and *CD79A* (**Figure 4B**). Monocytes in our data exhibited a reduction of pan-monocytic and known microglia-associated markers (*CD14*, *LYZ*, *TYROBP*, *CST3*). Macrophages preferentially expressed CNS border-associated macrophage genes (*NUPR1*, *IFI27*, *APOE*) (**Figure 4B**; **Supplementary Table S6**).

Next, we systematically compared the CSF cluster composition between disease conditions. We found a significant expansion of naive T-cell clusters in GAE compared with IIH and MS (**Figures 4C–E**). In contrast, monocytes decreased considerably in GAE during deterioration. To further explore whether dexamethasone induced a specific pattern of leukocyte changes affecting the cellular function of monocytes, we directly compared DEGs between GAE and the other two diseases (**Figure 4F**). We found that the gamma interferon-stimulated genes were still highly expressed in monocytes compared with IIE and MS after immunomodulatory treatment, including *IFNGR1*, *IFNGR2*, *TLR2*, *TLR4*, *JAK1*, *JAK2*, *STAT1*, and *MyD88* (**Figure 4F**). The expression difference was insignificant for other types of immune cells (**Supplementary Figures S6**, **S7**).

The pathways involved in the inflammatory and immune response to parasites in GAE were revealed by global functional enrichment analyses. As shown in **Figure 4G**, except for upregulated “phagosome” (*q*-value = 2.41*E*−22, **Figure 4G**), “tuberculosis” (*q*-value = 3.64*E*−06, **Figure 4G**), “leishmaniasis” (*q*-value = 3.64*E*−06, **Figure 4G**), “toxoplasmosis” (*q*-value = 3.64*E*−06, **Figure 4G**), and “amoebiasis” (*q*-value = 3.64*E*−06, **Figure 4G**), “Toll-like receptor (TLR) signaling pathway”, JAK/STAT signaling pathway, PI3K-Akt signaling pathway, and “NF-kappa B signaling pathway” (*q*-value < 0.05; **Supplementary Table S6**) were activated in monocytes. JAK/STAT signaling pathway and PI3K-Akt signaling pathway were also significantly enriched in CD4+ cells. According to a previous study in mice, depletion of CD4+ T cells led to high mortality and weight loss (25). Moreover, resident CD4+ T cells recruited inflammatory monocytes via CSF-1 as a mechanism against *Leishmania* in mouse infection models (27). Interestingly, we also found that *TGFB1* and *PRKCB*, which were considered dependent in anti-amoeba and anti-leishmania mechanisms, were highly expressed in monocytes (*q*-value < 0.05; **Supplementary Table S6**; **Figure 4F**).

## Discussion

In this study, transcriptome sequencing technology was used to show the different stages of infection with *B. mandrillaris* from the possible response mechanism. We mainly tried to explain the immune response mechanism in infection through the enrichment pathway and the upregulation of genes. It was found that the PI3K-Akt pathway in P1-CSF1 was highly enriched. The PI3K-Akt pathway is a very important intracellular signal transduction pathway that responds to extracellular signals and promotes metabolism, proliferation, cell survival, growth, and angiogenesis (28). Additionally, activating PI3K-Akt signaling is a strategy employed by viruses to slow down apoptosis and prolong viral replication in both acute and persistent infection (29). Indeed, we had also enriched some pathways related to viral infection, such as human papillomavirus infection. It is possible that *B. mandrillaris* infection shares similar mechanisms with viral infections. The TLR signaling pathway was the upstream pathway of PI3K-Akt, where the *TLR2* and *TLR9* genes were upregulated. *TLR2* activation first initiates downstream signaling cascades via the adaptors MyD88, TIRAP, TRIF, and TRAM, which polymerized on the PI3K and NF-κB pathways and regulate intracellular kinase and gene expression (30). *TLR9* binds to DNA present preferentially in bacteria and viruses and is normally activated by unmethylated CpG sequences in DNA molecules. It translocates from the endoplasmic reticulum to the Golgi apparatus and lysosomes and interacts with MyD88, triggering a signaling cascade that leads to a proinflammatory cytokine response, primarily inducing the production of type I interferons, IL-6, TNF, IFN-α, and IL-12 (30). *TLR9* is an important receptor expressed in immune system cells such as dendritic cells, macrophages, and other antigen-presenting cells (31). During infection, dendritic cells were depleted, which coincided with a decrease in *TLR9* expression.

In terms of the immune response, the depletion of monocytes and dendritic cells was mainly found. The factor of other types of immune cells in the process of *B. mandrillaris* infection has not been reported. Dendritic cells, as the most functional and specialized antigen-presenting cells, can activate the adaptive immune system after recognizing pathogen-associated molecular patterns (PAMPs) on infected cells (32), and they play an important regulatory role in the immune response. Monocytes are also very common immune target cells for many diseases. The depletion of these types of cells might contribute to the development and worsening of *B. mandrillaris* infection.

From the bulk RNA-seq data analysis, the content of IFN-α in P1-CSF1 could increase, while the content of IL-6, TNF-α, and IFN-γ did not increase. However, our results were just the opposite. Presumably, cytokines secreted by cells in other parts entered the CSF through some pathway, rather than through secretion by cells in the CSF. For example, after *B. mandrillaris* broke through the blood–brain barrier, it stimulated cerebral vascular endothelial cells to secrete large amounts of cytokines (4), which then entered the CSF.

*B. mandrillaris* infection is very rare worldwide, and the mortality rate is very high. It is very difficult to collect more cases for further validation experiments. The pathogenesis of *B. mandrillaris* infection has also been rarely reported, and our study represents a beginning for investigating this mechanism.

## Data Availability

The data that supports the findings of this study are available from the corresponding author upon reasonable request.

## Supplementary material

Supplementary Figure 1 The diagram of the “PI3K-Akt signaling pathway”

Supplementary Figure 2 The diagram of the “Toll-like receptor (TLR) signaling pathway”

Supplementary Figure 3 The diagram of the “JAK/STAT signaling pathway”

Supplementary Figure 4
**(A)** Volcano map of DEGS in P1-B1. A total of 1707 genes were up-regulated. There were 1958 genes down-regulated. **(B)** The pathway enrichment of up-regulated DEGs between P1-CSF1 and the negative control. The top one enriched pathway is the “PI3K-Akt signaling pathway”. **(C)** Changes in immune gene and ISG expression in P1-CSF1, P1-CSF2, and P1-CSF3 compared to the negative control.

Supplementary Figure 5 Innate and adaptive immune cell type and proportions in CSF among P1-B1, P1-B2, and P1-B3.

Supplementary Figure 6 Differential gene expression of various types of immune cells between GAE and non-infectious control (IIH and MS).

Supplementary Figure 7 Functional enrichment analysis of various types of immune cells upregulated genes between GAE and non-infectious control (IIH and MS).
