# Supplementary figures and images for: Bulk and single-cell transcriptome profiling reveals the dynamic immune response in granulomatous amebic encephalitis caused by Balamuthia mandrillaris: a cohort study
Source: Front Immunol. 2025 Oct 2;16:1677014. doi: 10.3389/fimmu.2025.1677014 (PMC12527871; doi:10.3389/fimmu.2025.1677014)

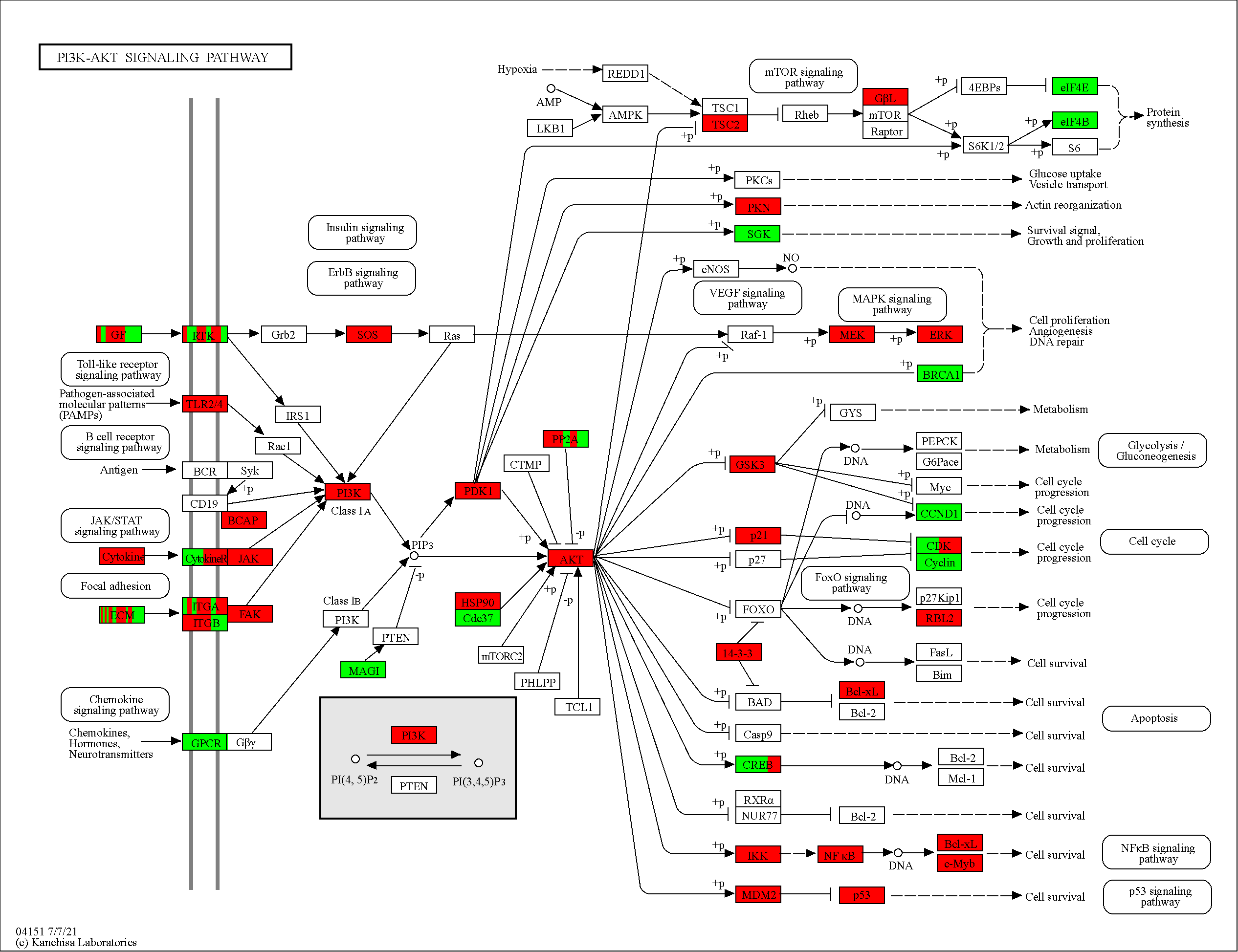

Supplement: Supplementary Figure 1 — The diagram of the “PI3K-Akt signaling pathway” [file Image1.png]

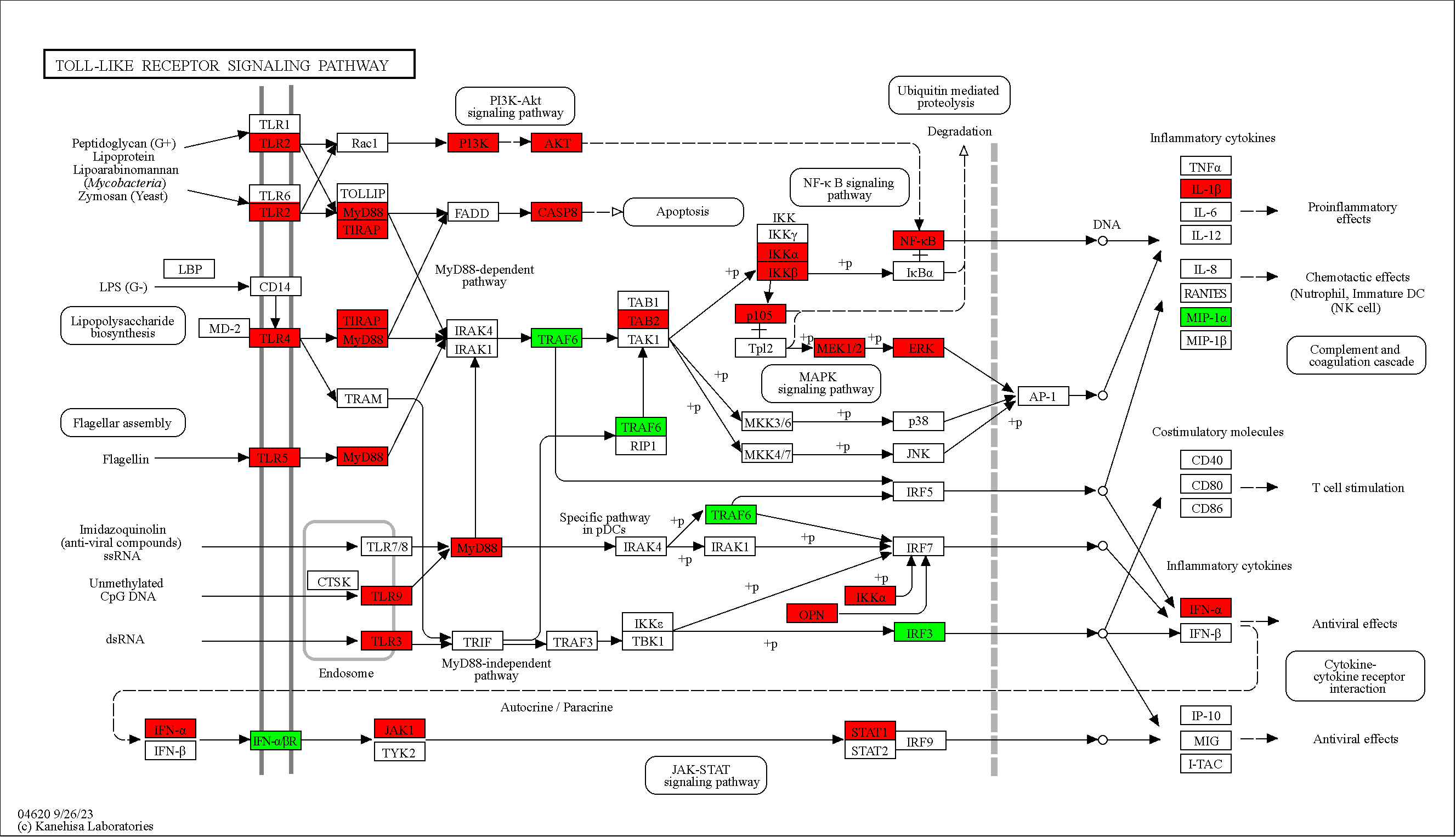

Supplement: Supplementary Figure 2 — The diagram of the “Toll-like receptor (TLR) signaling pathway” [file Image2.png]

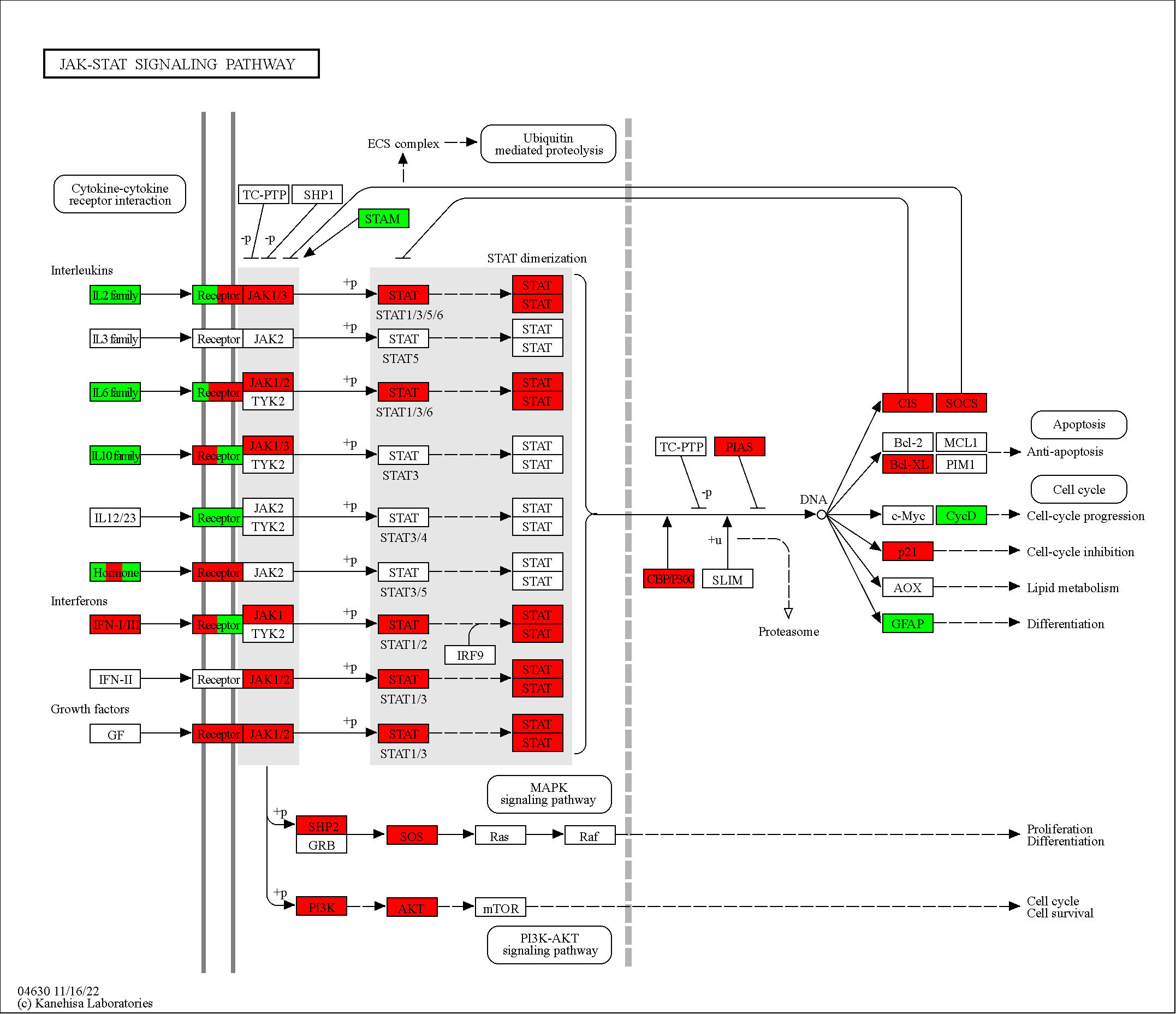

Supplement: Supplementary Figure 3 — The diagram of the “JAK/STAT signaling pathway” [file Image3.png]

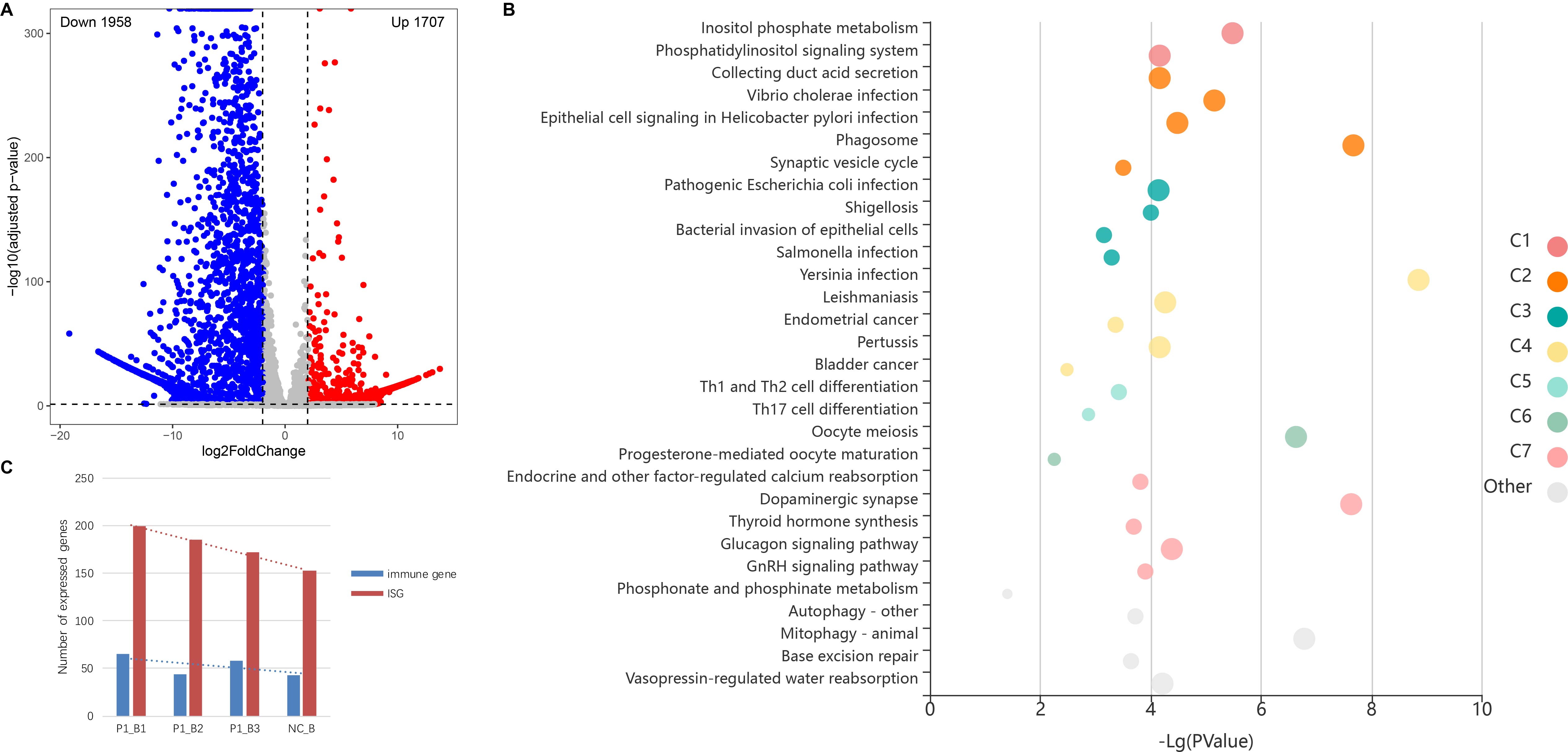

Supplement: Supplementary Figure 4 — (A) Volcano map of DEGS in P1-B1. A total of 1707 genes were up-regulated. There were 1958 genes down-regulated. (B) The pathway enrichment of up-regulated DEGs between P1-CSF1 and the negative control. The top one enriched pathway is the “PI3K-Akt signaling pathway”. (C) Changes in immune gene and ISG expression in P1-CSF1, P1-CSF2, and P1-CSF3 compared to the negative control. [file Image4.jpeg]

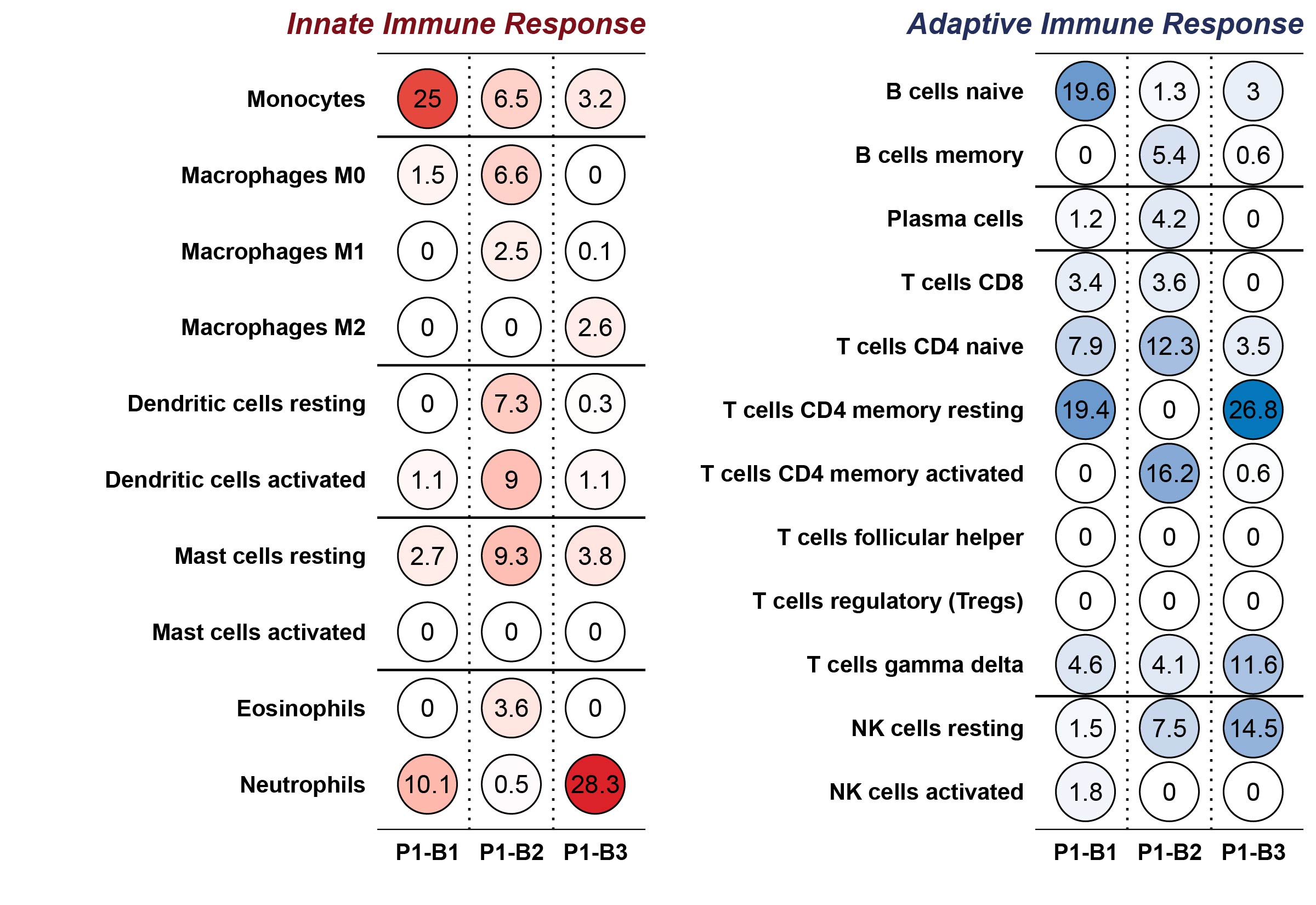

Supplement: Supplementary Figure 5 — Innate and adaptive immune cell type and proportions in CSF among P1-B1, P1-B2, and P1-B3. [file Image5.jpeg]

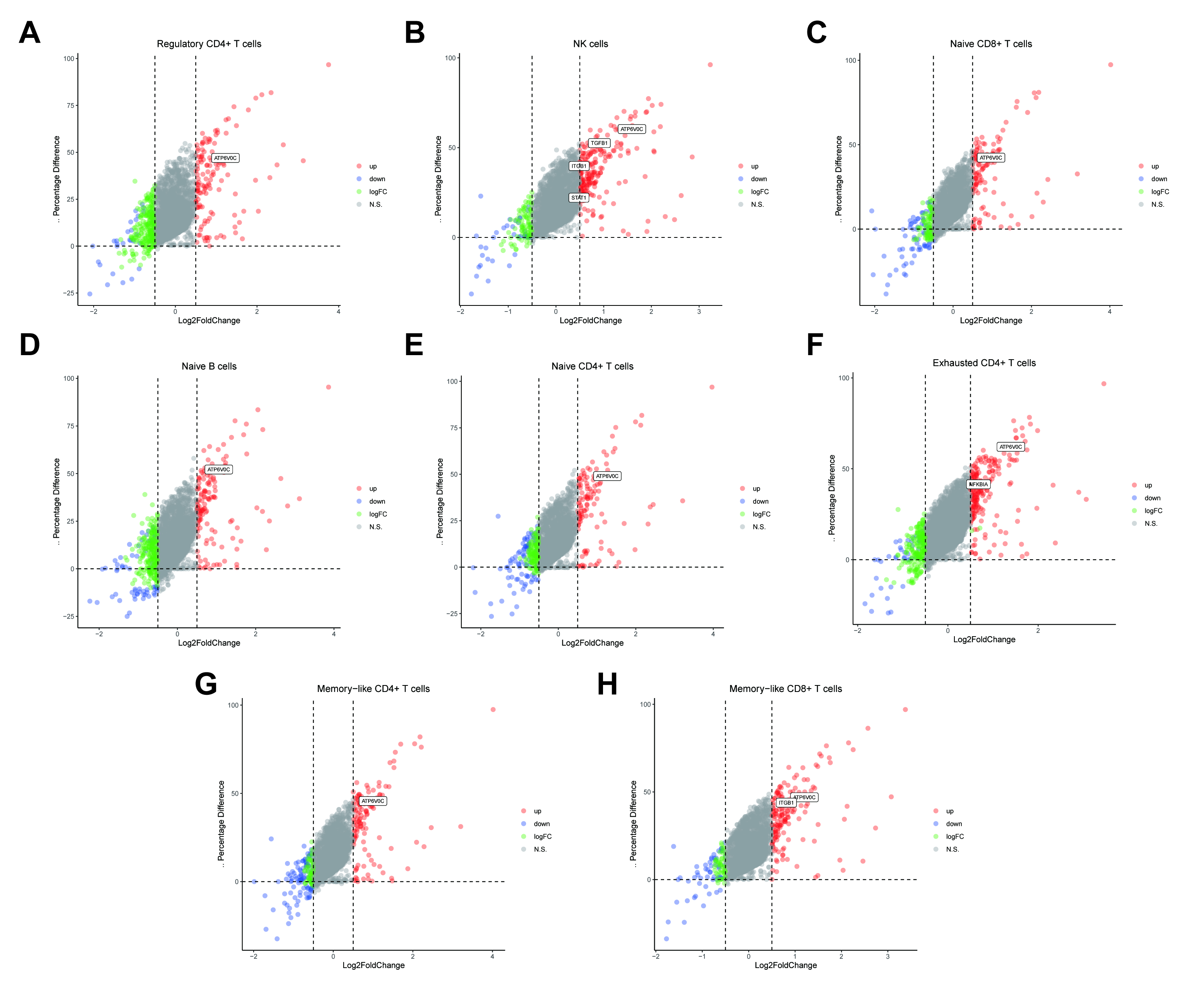

Supplement: Supplementary Figure 6 — Differential gene expression of various types of immune cells between GAE and non-infectious control (IIH and MS). [file Image6.tif]

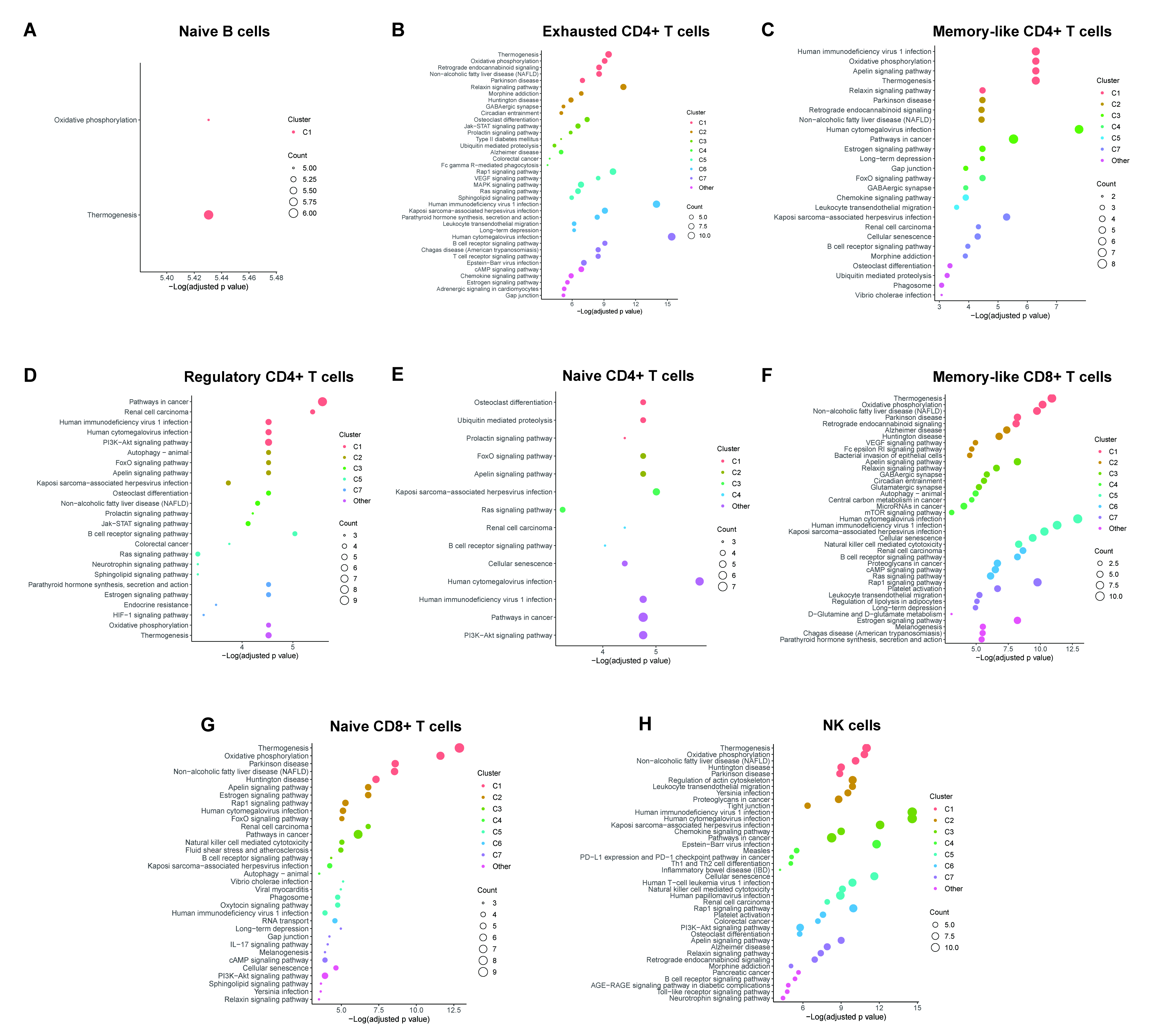

Supplement: Supplementary Figure 7 — Functional enrichment analysis of various types of immune cells upregulated genes between GAE and non-infectious control (IIH and MS). [file Image7.tif]
